# Supplementary material for: Transcriptome analysis of flavonoid biosynthesis in safflower flowers grown under different light intensities
Source: PeerJ. 2020 Feb 21;8:e8671. doi: 10.7717/peerj.8671 (PMC7039124; doi:10.7717/peerj.8671)
Supplement: Supplemental Information 1 [file peerj-08-8671-s001.docx]

| Gene Name | Specific Primers (F: Forward, R: Reverse) |
| --- | --- |
| *CtHCT1* | F: CATCTATGTTACCCTCCTATT  R: ACTTGCCAACTGCTGTAT |
| *CtHCT2* | F: GCTCTCGCACTCTATCACCC  R: TTAGCCGAATACCCGTCTGC |
| *CtHCT3* | F: TCGTCTTCTACTACCCTAACC  R: GGCAACAGCCTATTCAGC |
| *CtFLS1* | F: TCCACCTTGTCCAGAGCC  R: TCCGAGTTCACGGTTGCT |
| *CtFLS2* | F: GAGGTTCATTGCCTTCCA  R: AAGTTCTTGCCGTATCCC |
| *CtANS1* | F: GAGCCTGACCAAGTCATGGG  R: TTTCCGAGTTCACGGTTGCT |
| *CtANS2* | F: CGATATCGCATCCGGGAACA  R: GAGTATCTCGGTGGCGAGTG |
| *Ct25S* | F: GGAGGTTGAGGGAAAAGGAG  R: GTGACCTCGTCACCCGTAGT |
