# Supplementary material for: Transcriptome analysis of flavonoid biosynthesis in safflower flowers grown under different light intensities
Source: PeerJ. 2020 Feb 21;8:e8671. doi: 10.7717/peerj.8671 (PMC7039124; doi:10.7717/peerj.8671)
Supplement: Supplemental Information 2 — Total Raw Reads (Mb): The reads amount before filtering Total Clean Reads (Mb): The reads amount after filtering Total Clean Bases (Gb): The total base amount after filtering Clean Reads Q20 (%): The rate of bases which quality is greater than 20 value in clean reads Clean Reads Q30 (%): The rate of bases which quality is greater than 30 value in clean reads Clean Reads Ratio (%): The ratio of the amount of clean reads. [file peerj-08-8671-s002.docx]

| Sample | Total Raw Reads (Mb) | Total Clean Reads (Mb) | Total Clean Bases (Gb) | Clean Reads Q20 (%) | Clean Reads Q30 (%) | Clean Reads Ratio (%) |
| --- | --- | --- | --- | --- | --- | --- |
| HL_1 | 124.51 | 110.94 | 11.09 | 96.57 | 88.41 | 89.1 |
| HL_2 | 122.02 | 109.49 | 10.95 | 96.7 | 88.73 | 89.73 |
| HL_3 | 122.02 | 110.59 | 11.06 | 96.66 | 88.6 | 90.63 |
| LL_1 | 122.02 | 109.31 | 10.93 | 96.69 | 89.03 | 89.58 |
| LL_2 | 122.02 | 111.23 | 11.12 | 96.87 | 89.22 | 91.16 |
| LL_3 | 124.51 | 110.7 | 11.07 | 96.56 | 88.44 | 88.91 |
| ML_1 | 119.53 | 109.51 | 10.95 | 97.3 | 89.67 | 91.62 |
| ML_2 | 119.53 | 109.1 | 10.91 | 97.22 | 89.49 | 91.28 |
| ML_3 | 122.02 | 110.84 | 11.08 | 96.99 | 88.73 | 90.84 |
