# Supplementary material for: Transcriptome analysis of flavonoid biosynthesis in safflower flowers grown under different light intensities
Source: PeerJ. 2020 Feb 21;8:e8671. doi: 10.7717/peerj.8671 (PMC7039124; doi:10.7717/peerj.8671)
Supplement: Supplemental Information 3 — Unigene31654_All, Unigene18001_All, CL1709.Contig9_All, CL10633.Contig6_All, CL3969.Contig2_All, CL10633.Contig3_All and CL9672.Contig4_All are named CtHCT1, CtHCT2, CtHCT3, CtFLS1, CtFLS2, CtANS1 and CtANS2 respectively. [file peerj-08-8671-s003.docx]

| Gene ID | Group(s) | Log2FC | Regulated | GO annotation | KEGG annotation |
| --- | --- | --- | --- | --- | --- |
| Unigene31564_All | HLvsLL/  HLvsML | -2.66633258/ -2.92618536 | Down/  Down | NA | egr:104421208/3e-14/omega-hydroxypalmitate O-feruloyl transferase; K15400 omega-hydroxypalmitate O-feruloyl transferase [EC:2.3.1.188] |
| Unigene18001_All | HLvsLL/  HLvsML | -2.15245172/ -1.97822422 | Down/  Down | NA | mtr:MTR_8g096530/5e-28/omega-hydroxypalmitate O-feruloyl transferase; K15400 omega-hydroxypalmitate O-feruloyl transferase [EC:2.3.1.188] |
| CL1709.Contig9_All | HLvsML | -3.265573297 | Down | NA | jcu:105635402/9e-94/vinorine synthase-like; K13065 shikimate O-hydroxycinnamoyltransferase [EC:2.3.1.133] |
| CL10633.Contig6_All | MLvsLL | 2.313843715 | Up | NA | vra:106769199/5e-51/flavonol synthase/flavanone 3-hydroxylase-like; K05278 flavonol synthase [EC:1.14.11.23] |
| CL9672.Contig4_All | HLvsLL | -1.542934946 | Down | NA | fve:101296827/5e-41/flavonol synthase/flavanone 3-hydroxylase-like; K05278 flavonol synthase [EC:1.14.11.23] |
| CL10633.Contig3_All | MLvsLL | 2.571737817 | Up | NA | pda:103696959/2e-65/leucoanthocyanidin dioxygenase-like; K05277 leucoanthocyanidin dioxygenase [EC:1.14.11.19] |
| CL3969.Contig2_All | HLvsLL | -1.846834147 | Down | NA | vvi:100233142/1e-156/LDOX, ANS; leucoanthocyanidin dioxygenase; K05277 leucoanthocyanidin dioxygenase [EC:1.14.11.19] |
| Unigene15127_All | HLvsLL/  HLvsML | -3.03051123/ -2.50293348 | Down/  Down | NA | mtr:MTR_7g086000/5e-09/transferase family protein; K13065 shikimate O-hydroxycinnamoyltransferase [EC:2.3.1.133] |
| Unigene18529_All | HLvsLL | -3.76674 | Down | biological_process:GO:0009813//flavonoid biosynthetic process;GO:0052696//flavonoid glucuronidation;cellular_component:GO:0043231//intracellular membrane-bounded organelle;molecular_function:GO:0080043//quercetin 3-O-glucosyltransferase activity;GO:0080044//quercetin 7-O-glucosyltransferase activity; | egr:104456188/3e-55/UDP-glycosyltransferase 74F2-like; K13691 pathogen-inducible salicylic acid glucosyltransferase [EC:2.4.1.-] |
| Unigene62812_All | MLvsLL | 6.465383158 | Up | NA | cam:101510753/2e-58/phenolic glucoside malonyltransferase 1-like; K13264 isoflavone 7-O-glucoside-6''-O-malonyltransferase [EC:2.3.1.115] |
| CL285.Contig2_All | HLvsML | -2.925180781 | Down | NA | mdm:103439955/6e-20/caffeic acid 3-O-methyltransferase; K13066 caffeic acid 3-O-methyltransferase [EC:2.1.1.68] |
| CL10851.Contig1_All | MLvsLL | 6.53575614 | Up | NA | spen:107014577/0.0/shikimate O-hydroxycinnamoyltransferase; K13065 shikimate O-hydroxycinnamoyltransferase [EC:2.3.1.133] |
| CL18920.Contig24_All | HLvsLL | 4.460283 | Up | biological_process:GO:0009813//flavonoid biosynthetic process;GO:0052696//flavonoid glucuronidation; cellular_component:GO:0043231//intracellular membrane-bounded organelle; molecular_function:GO:0080043//quercetin 3-O-glucosyltransferase activity; GO:0080044//quercetin 7-O-glucosyltransferase activity; | vra:106769098/1e-33/UDP-glycosyltransferase 83A1-like; K04628 ceramide galactosyltransferase [EC:2.4.1.47] |
