# Supplementary figures and images for: Transcriptome analysis of flavonoid biosynthesis in safflower flowers grown under different light intensities
Source: PeerJ. 2020 Feb 21;8:e8671. doi: 10.7717/peerj.8671 (PMC7039124; doi:10.7717/peerj.8671)

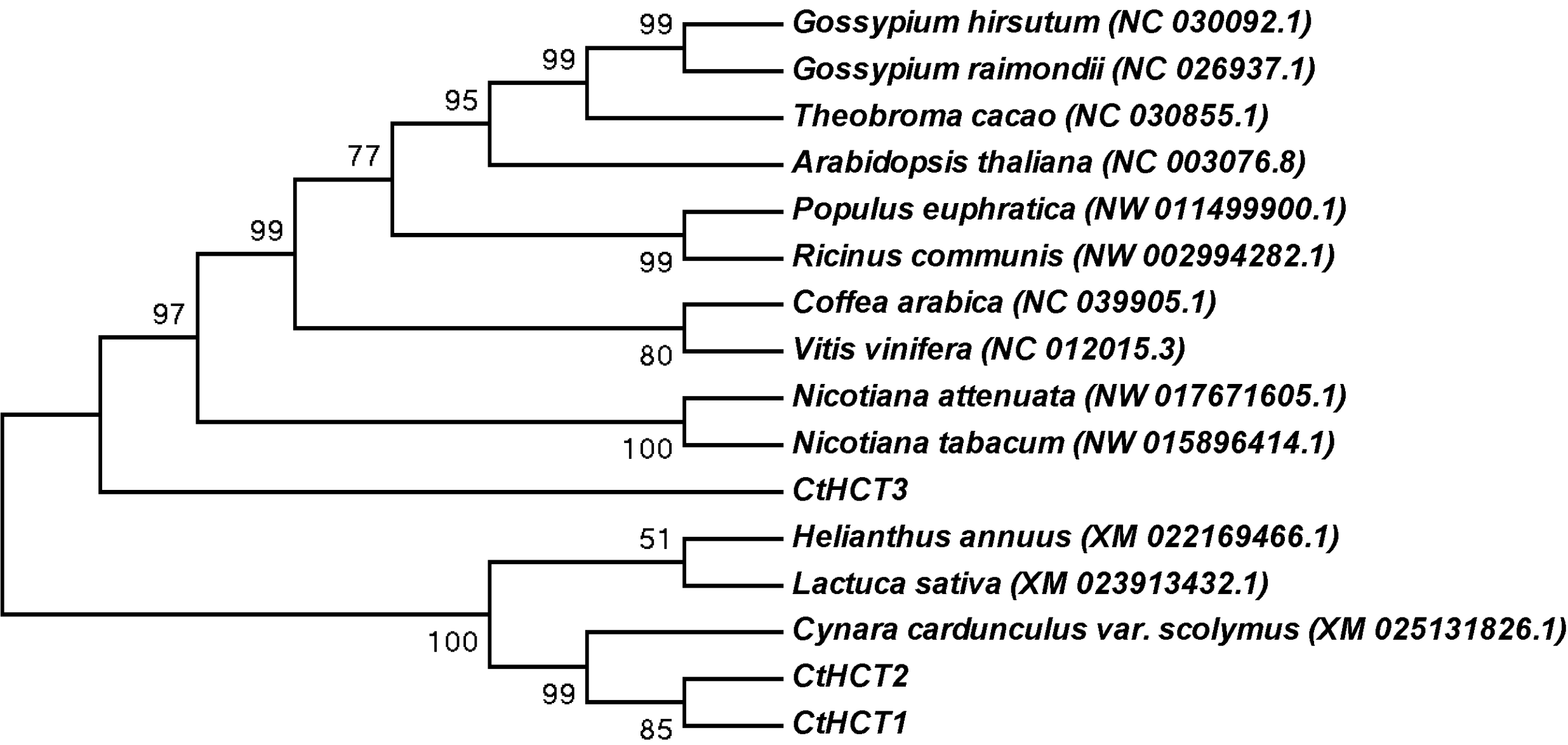

Supplement: Supplemental Information 4 [file peerj-08-8671-s004.pdf]
