# Supplementary material for: Transcriptome analysis of flavonoid biosynthesis in safflower flowers grown under different light intensities
Source: PeerJ. 2020 Feb 21;8:e8671. doi: 10.7717/peerj.8671 (PMC7039124; doi:10.7717/peerj.8671)
Supplement: Supplemental Information 5 — Unigene31654_All, Unigene18001_All, CL1709.Contig9_All, CL10633.Contig6_All, CL3969.Contig2_All, CL10633.Contig3_All and CL9672.Contig4_All are named CtHCT1, CtHCT2, CtHCT3, CtFLS1, CtFLS2, CtANS1 and CtANS2 respectively. [file peerj-08-8671-s005.docx]

>Unigene31564_All

GCTAGAAATATATAACACATATGAAAGAACTTTTATTGGAACATGAACTATTAAGTAATCTAACGTACGATATACAATTCGCACATCTTAGTAACTTAAGAGCAGATGAGTAACTTCATATTTTCTAAATTAAACAACATCAATCAAACCAATACAAAGAGTTAGATCTCGAGGTAATCCGCATTCACAGGCTTGAACATGTGACTTGCATCATGTGCCTCAATGTAGTTGATATGTTTGATCGGCATATGCATGTACACGACCCAATCCTCGCTGCTGGTGATCGGGCCAGGCAATGTCATCACGTAACAGTCCTTCCTCGTTGGTGGAATATAGCATGATCCGAAAGCCACCTTACCCCACCCAAAGTCCATATCGTTCATGAACTGGAACCTTTGTCCAGATGACACGATCACCGTCATGTCTTTGTTGATAAATGGTCTTGGTATTAGTACATGCGACCGTCGTTCTTCCACCCAGTTTATGATGTCTAGGAAATGTTCTTTACCGTTGACGATCTGCAAGAGCTCATGAACCTCCGTAGCTACATTGCTCAATGACATTTCAACCAACTCCTGTGATCCTTTTCCCCCAAATGGCATTGACAAAACATTGCCAAAGTGCGAAGCCATTAGCTTTTCTATCTGTTCTCCATCTCCTTCACTTAACCTTCGCCTTCCATCAACTGCAACAGCAACGTTACAATACCTCTCATTATGATTTCCCAATTTTTCCATGCTCATGCCAATTGTCTTCCACAAGAAAGATGTGAAGGCCTCAAACTTCGACCTCTTACTTCCATTTTCACTTGCCAACTGCTGTATCTTGTTAATTTGCTCACCCTTGATGTAGTATATACGGTTAATGAGTAGATCCTCGGATCCATCATTGTGGTTTTTATCATTATCTGGCGGCATGAATGATGGTTCAAATGTAGCAACGAAGTCGTCTATTGATGGCGAAGAACTATAAGTAGGTGGGCTTCGTGGTTTGAGGATGGACCTCGAATAGGAGGGTAACATAGATGGGATTTGGGATCGAGCCATGTCTGCCCATGATGAGATAAACATGTTAGCCGAATACCCGTCTGCTGCCTTATGATCAAACATGCATCCTATCACCATGCCTCCG

>Unigene18001_All

TGGTAGGGACATATATAGCTTAATCATGAAAGATATCAAAGAGGACACACACAGAGTACATTTTTAATATTTTAACAAGAAACAACTTGCAGATTTTCAAAAGTAAAACACATGAATTGAACCGAGACAAAGCGTGTTAAATTTTGAGGTAATCGGCATCCACGGGCTTGAACATGTGACTGGCATGTGCCTCGATATAGTTGATATGTTTGATTGGCATATGCATGTACACGACCCAATCTTGGCTGTTGGTGATGGGACCGGGCAATGTCATCACGTAACAGTCTTTTCTTGTAGGGGGAATATGGCATGATCCGAAAGCCACCTTACCCCAGCCGAAGTCCATATCGTTCATGAATTGGAACCTTTGTCCAGATGATATGATCATTGTCATATCTTTGGTGACAAATGCTCCTGGCAATAGTAGACGTGATCGTCGTTCTTCCACCCAGTCTATTAGGTCCAGGAAGTGGTCTTTCCCGGCGACGGTGTGCAAGAGCTCGTGAACATCTGTAGCTACACTGCTCAATGACATTTTAGCCAAATCCTGTGATCCTTTCCCCCCAAACGGCAATGTCAAAACATTGCCAAATTGCGAAGCCATTAACTTTTCTTTCTCTTCTCCATCTCCTTCACTTAACCTTCGCCTTCCATCAACTGCAACAGCAACGTTACAATACCTCTCATTATGATTTCCCAATTTTTCCATGCTCATGCCAATTGTCTTCCACAAGAAAGATGTGAAGGCCTCAAACTTCGACCTCTTACTTCCATTTTCACTTGCCAACTGCTGTATCTTGTTAATTTGCTCACCCTTGATGTAGTATATACGGTTAATGAGTAGATCCTCGGATCCATCATTGTGGTTTTTATCATTATCTGGCGGCATGAATGATGGTTCAAATGTAGCAACGAAGTCGTCTATTGATGGCGAAGAACTATAAGTAGGTGGGCTTCGTGGTTTGAGGATGGACCTCGAATAGGAGGGTAACATAGATGGGATTTGGGATCGAGCCATGTCTGCCCATGATGAGATAAACATGTTAGCCGAATACCCGTCTGCTGCCTTATGATCAAACATGCATCCTATCACCATGCCTCCGCATTTTAATTCCGTAACCTGGACAGCAAGCACCCCACGTAGCCTCTCGGGTACTAGTTTACCCTCAATGCTCTCATCCGGATTGTAGAAATCCAGTTCCTTCAGCTGCACGTCAGCAACGGCATGAGTAAAATCAACACCTTGGTTGTTGCAGTGGATCTGATTCTCTCCGGCAGCCGCATTCCAGGCGATCTCACCGGCGAGCGGGTGATAGAGTGCGAGAGCTCGCGATAAGGAATCTTTAAGGGTGTTGAGCATGGTGGTGAAACTACTACTACTCCCATGGGAGGGTAAGGGTTTCTTGTAGCAAAAGAAAGAAGCAGCATCAAAAGCAGGGACAACCAAATCCAGGTTTGTAAAGGATAGCCAGCGATCATCCCATGGCTCTTCTGCAGCCACCACCACCTTCTCAATCACCTTCACTGAGAAAGGCATACCTCTGATACCCATATTAATTAATTTGCACACAGAAAGAGAGAAGTGTGTGTGTGAGAGAGAAAGAGAGAGAGATCTAGAGAGAGGTG

>CL1709.Contig9_All

CACAGACTATAGCAACAAAGAAATCTCCCTCTAAACATACAAGAAAAATGAAGTGCATTTATAAACAACAAAGGCTACACGACTAAGCCACTACTTCTACCACTAACTATTTAGACCTCAAGAAAAGGACATCGTGACGAACAAAATAGAAAATACGCTTTCAACATAAATAAACGAAAACCCACCGAGAGAATCTGGATTAAACCAAATAGATGAAAATTAAACTTTATTTATTTTGTCAACGTTCGTAGAGGGCTTGGATCCACGGAAGCATATGCTAGTAGCTCCGAGTTGCCTTGTAGAATCTCCATCTCTTGTTCATCAAAGTTCACCCATGCTTCTATTCCTCCAACAAATGTTGTGTCCATTAGGGTGACCTGATTGACGAACACCGGGATGCCATGGTTGATGAAACCACTCACCCAACTCGGTTCTCCCCAACCAAAATCCATTTGGTAAAAACCCATCTTGCACCAACTTGTAAAAATGTAGTTATCCATGGTTCCTTTGGAGCTAATTTCTCCCATTTCTTTGATAGACTCCTGCATCGCAAGGTACCCCTTTTCACCTTGTGCTTTGTTCACAAATTCCATGTCGATTTTGGAAATGCTTTCATGCACCTTTTCCACCAAGCCATGCAATGTAATCTCATAGCCAGCTTGGCACTTTGCGGTTGCTGGCCAAATCACATTACCGATGGAGTCTTTTGGTAAGGTTGATGCGAGTTTTCTGCGAAGGTTCACCATGTGCGTTAAGCGAGAAGGTTTCTGGAGACCAGTGGCTTGTTTGGATGCTGCCAGTGCACACTTCCATATCAAAGCAGTTACCACCTCAACGCTTGTAGGGTTCTTCACTCCATTCCGTGTTGCATTAGCTTTCAATCTCGCTATTGAATCAGAATCAAATACAAATCTTTTCGTGCTACATTTCCCCTCCTTTAACCACGACCCACACATGGCCATTGAAGATTCTTTAAGCCACAAGTCTTTCGCAGGGAAGAGAGAGGGTGCAGTAAAGTTTGGATGCACGACTTCTTTAGCTCCACAAGCCACGTCGGTCCATCCTCTTAGAAAAGTGTATAGCGAGGCCCCATCAACGACCTTGTGTGAAACACACAAGCTGATAGCGATCCCACCACATTCGAAGATGTTCACTTGAACATTTGTTACACGAGCACCTACACAAGACCGCTCGAAACTAGGTTGAACCGGCAACAGCCTATTCAGCTGCTCGTGGTCAGGATGGCTCAGAAGCTCGTTAAGACGACAACGAACCAAAGCTATGGCATAGGAAGCACCAACGTCATTGCAGTCGATGGAAAGATCATCCTTGATGGTTCCAGCGAGAGGGTAAAACCGAGTCAAGGTTTTGGATAAAGATTTCTTCAAGGCTAGTGCTCTCTCTAAGGCTTGGAGGATGGTGCTGCCATTGTGGTTAGGGTAGTAGAAGACGATCGGTACATAAGGATCCATAATAAGCTGATCTAAGAGGGAGAGGTTGAATGTTTTCAAGTGGTGTGGCGTAGGGGAAGATGGTTTGATGCTCTCTTTGGAAATGATTTCAATATTCATTTCTTAAAATTTTGTTGGAACTTGGAAGTGTAGATCAGATAGATGGTTTTTGTGGAAGATGACTTTGTATGTAGAATAAGATGATTGTTGTATAAGATATGTGAGGCTGAAGAGCCTTTTATAGTGAAGGTATGAAGACAGATTTCATAG

>CL10633.Contig6_All

GGCTTATCTGTCGTTTCACATTACTTGTATTTGAAACATTTTGTTTAATTTGGCTGTATTTGTTAAAGTCCATTGTTTGAGGACTTAAAGTGAAAATTGTGTGATGACTTTGTGGGCTATATAATAGCCTTACCTTTCTAGGGTTTGTCCCTATGAATGATAAGTGATATTTTTGGTGGCTGAGATATTGTGTGAACATAGCTTTCCTTATAGTCATCTTCTTCTTTGCTTGTCATCTTCATCACAAGCTTGTGCTTACTTTCTCTACAAATCTTTGTCAAGCTTATCTCATCTTGTTTATCACTCTCTTTGGGACTCACAGCTAAATAAGATTGAGTAAGGGCATTATATATAGATATCGATAAAGACTTAATTAGGATGTTGAACCGATAATATAGGACTTACCTTACTTATGAATTGTTTAGGGCACTTTTAGGCTCGGGGTTGGAATTCAAATCGTTGTTCGAATCGCTAAGCTTACCAAGGTGAGTATTCACAGCCCCTCTTTTATCGATTTTACGTATTTGGGGTGAAAAGCATGTTGATTGATGAAATATGTTATGAGTTATGCTTACGAGCAAATTGTTATGCAAATGATGATATGAATTGAGTAAAAGTCCCCTTATAATATACACTACTATTAACTCATTAGGAGCCTATGATCCAGTGGATAGCGCTATTTGGTAAAGAGACTCACCCTTCTGACTGACGAGTAGAGGGTGCACTATTTCAGAATTAACGACTGACCGCTGAGGTAGTCGTTGGGAAACGTTCTACACATGTGGGGTTCTAGTCCCACCGCTGAGGTAGTTCATAAGAGCGTTATACCCTACGTGGTTCGCGACCGCCTCTGAGGTATAACGTTGGGGTGTTACGGTTTGTAGTGGCTCAATCTTTCATATCAAGTGCATAAATAACGTTATTTAACGTTTAATGATTGAACAAAGGATTTTCGATAATGATTTCTGCATATGAAACTGTTTTGTGCATATAAAACCTGTGAACTCACCAACTCTTGTAGTTGACACTTGTTTTAACATGCTTTTCAGGAAAATAGAAGGTAGTTGAATATGCCTGTTGTTGCAAGTGACATCAATCTTCAGACTTTTCTTTTCTTTTGTTATTTTGGATGTTTGAAAGTTAAACTATCGTTGGTTATGTAATATGTGGTACTGTAAACAATGAACTTTTAAGTAAAATGATTGATGTTATTTATCTCCTATCGCAATGAAATCTTAGCTGTCACGTCTCGCGTTTCCGCTTTAGCGGGGTGTTACACTCATCAAGTCATAGAGGAGTTGGACCAACACTATCTCATTCAAAGCATACCCAATGAGATCTATGCAAACATTGACTGCAACGACACGGGGAAGGCGATGTGGGATGAAATCTGTAGTCTGATGCATGGTACGGAGAAGGGTATTCAGATGAAGAGATCTAACCTGTTAACAAAGTTTGCCACCTTCAAAAGTCGTGAGGGGGAGTTACTTGAAGAAACCTACCATAGGTTCTGCACAATAATAAATGAGTTGAGGAAGAACAAGCTCAAGAAGTCTCATTTGGAAATCAACATCCAATTCATCAACTCACTGCGATCTGAGTGGAGAAGATATGCCTTAAACATTCAACAGAATCGCAGCCAGGAAGATATAGACAGTCATGAACTGTTTGAGTTACTTAACCACAACCAAGATGAAGTGCTTGAAATACTTGGTACTGAGATGAAAGTAGAAAAATATGTTGATCCACTTGCCTTGGTCGTTGACAAAAGACAATACTTGGTACTGAGATGAAGTGCTTTAAAAAGTTATACTTTTTTAGATCTATAGTTGCATTCAGGAAAAGAAGTTATTTTTTTTGGAAGGGTGCCCGGGCCAAATACTGACTAATCCCCGACAATTTTTGGAGGCTAAATATGGTCCCACTTAAATCATAAAAGCCATTTTAAGTAGACAAAGCATACGGTCATTATTTATATGATACACACACATTGTTTTTTTAATACAGACCTAGATATAATAAATCATAGATAGTATTGCTTAAGGTGCGATTTGCTCTTTAGTTCCTTCGAAAAAAAGTTCTTCAAGAAATCTGCAACACTTACTCTTGTAAATCTTGGAGGTGTAGCAGCAGTGATTAGGCTTGGAGCTGGTCCCAAATCACCTTCCAATACAGGTCCTAAAAAAGTGGCTATTGAAAGCCTCTCCTTTTCCGAGTTCACGGTTGCTCTATGCTCTATGCTTTTATATATTCCGTTTGTCAGAATCTCCAAGACGTCTCCAATATTGACAATGAAGGCATCATTCAGTGGTGTAACTGGCATCCAATTGCCATCCTTTTTTATTTGGAGACCTTCTACTTCATTGATTTCGAGGAGGAATGTGATTCCATGAGCATCTGAGTGAGGGCTAAGACCCATGACTTGGTCAGGCTCTGGACAAGGTGGATAATAGTTCATCCTCATAGATTGCCATCCCTCATCAAATAATACTCTCATATCCTCAACCTTCATCTTTGAAGCTTTTGCAATGAAGATAAGAGTTTTTAAAGCAAGGCACTTCATTTCTCTCGAGTACTCTTCTAGGGTATCTCTGAATGGAAGAGGTAGATTGGGAAATAAGTTTGGCTTCCTCAGATGATGAGGAAGGGTAATCATGTAAAACATGTCAGCCCAATCAAGCTTTTGCTCCTCAGATACAACAAAAGCCTGTCCAAATCCTTGTATGTCTCCTTCATCTTGCCAAAAGCTCTTTTTCTTTTCCATTGGTAAGTTGAAGAATTCTTGTGTTTCTTCCTTCACTTTCTCCAACAATGAACAACTCACCCCATGATTTATCATCTGAAAGAAGCCCCAATCTTTACAAGCAAGATGTAGTTTTTCCAATTCATCATTACATGATTCCTTGGACGCTAATCGTTGCATATCGATGACGGGTACTTTAGTCATTGAAGGCAATAACCTTGAAATGGCAGGAGGGCCATCATTAGGACGAACGTATCTGGATGGAATCTTGGTTAGTGGTTTCTTAGCCAGTTCTTGGACGCTCGGCACCAAAAGTGAAGCCCAATGGCTTGTTACATTGGGTTCCATGATACCTACCTCTAGACGTACTCTCTCCCTTTAATTTGTTATGCTTTTTTACATGTATCTCCGCATTGTTGCTAGAAATATATACACACGTCGATCAACACGTGTTGTTGATACGTATCGGGTTTGAAAG

>CL9672.Contig4_All

CCCTATGTCTAGTCTTGTGTCGATGTTGTTAACGAATCTTGACCACAAATTCGTATCATAAATATATTTAAAACAGCCAAATCGCTCTAAATCCCCAATTAATCATGTCTATCAATGAAGGTTACGATGGTCCTCACTCTCATGAAGTCAGTTTTATCGACGAAAATGGTGAACTTAAAACCACCAAGATTCCTGTGGTTCAAGAGCTTGCACGCCAAGGCCTCACCAACGATCACTTCCCCAAGAGGTTCATTGCCTTCCAATCAACAACTTCCGCTGTCGATTGCCAATCGGTAACCCCTCCGGTGATTGACGTAGTGAAACTAAAAAGTGATGTAACCCGAGGGTTGGAGCTGCGGAGGCTGGCCGATGCTGCCAAAGAATGGGGCGTGTTTTTGGTCAAGAATCATGGGGTGGATGATACGGTGTTGGATGATGTTAAGGATGTTGTGAAAGGTTTCTTCGGGTTGGGTTTTGAGGAGAAGAAGGCCAATGTTGGATCGTATAAGAGCGTGGATAACATGGGATACGGCAAGAACTTCGTGAAGTCCGAAGATCAACCGTTGGATTGGATCGATCGGCTCACCATGAAAGCGGCTCCTGTGGATCCAGATGAAGCAACTAATGGCCTCTTAATTTGGCCTAGAAAACCAACAAACTTCAGGAAAGCCGTAGAAAAATATGTGGAAAAATCAAGAAAAATTCTCGATGGGTTACTCCAAGACCTTGCAGAATCGCTATCACTAGATAAAAATGCTTTCTTGCAACAGTTTGAGCCAAAAGAAAGTGAGATCAAGGTTAGGGTGAATTACTACCCGCCGTGTCCAAGACCGGACCTGGCCATAGGAATCATGCCGCACACCGATCCTAGTGGCCTCACGCTCTTACTCGAGTTCGGAACCACAAGTGCCCTCCAAGTGCAGAAAGACAACTTTTGGACCACCCTTCAATTGCCGAACGATAACAGTTTGGTCGTCAGTATCGGAGATCTGCTCGAGATCATGAGCAACGGCATGCTGAATAGCCCGTGGCATCAAGTGCGAACACAGCTCGAAATGGAACGGTTTTCACTGGCCTGTTTTTATAACCCGCCGGCCAAAAGTGAAATTGGGGCGGTGGTCGGAGGTGATTCATCGGAGGAGATATATAAGAAGGTGGTGGTGGAGGACTATGTAACTAACTATTACAAAATTAGCCCAACAACTAGCAAGGAGGCAATAATGGCTTTTCTTTTCATTTTCCGTTTGGAGAAAACCTTTATTGTGTGCTTAATTGTAATTTTAAAAGTTTGAAAAAGTTCTTTCGATCATAAACGTTATTGGAATTAGACGATGGGTAATTTTATATATCCACCTATGCAGTCATTGGTTTGTACAATTGTAATTTTTATCTCATATATATTGTCTCAATCATTT

>CL10633.Contig3_All

GTTATCTTCTGCCATCCCCTCATGCTTTCAGTATTAGAAAACGACATATAACAACATAAAGAATGGTTCAATAACTTAAGCTCCTTTCGAACCCGATACCTATCAACAACACGGGTTGATCGATGTGTTTATATATTTCTAGCAACAATGCAGAGATACGTGTAAAAAAAAACGTAACGAATTAAAGGGAGAGAGACATCTATAGGTATCATGGAATCCAATGTAACAAGCTACGGGGTTTCACTTTTGGTGCCTAGCGTCCAAGAACTCGCTAAGAAACCACTCACCAAGGTTCCATCCCGATACGTTCGTCCTCATGATGGCGCTCCTGCCATTTCAAGGTTGTCGCCCGCAATGACGACGAATGAACTACCCATCATCGATATGCAGCGATTAGCGTCCCAGGAATCATGCAATGATGAACTGGAAAAGCTACATGTTGCTTGTAAAGACTGGGGTTTCTTTCAGATGATAAATCATGGGGTGAGTTGTTCATTGTTGGACAAAGTGAAGGAAGAAACACAAGAATTCTTCAACTTACCAATGGAAAAGAAAAAGAGCTTTTGGCAAGATGAAGGAGACATACAAGGATTTGGACAGGCTTTTGTTGTATCTGAGGAGCAAAAGCTTGATTGGGCTGACATGTTTTACATGATTACCCTTCCTCATCATCTGAGGAAGCCAAACTTATTTCCCAATCTACCTCTTCCATTCAGAGATACCCTAGAAGAGTACTCGAGAGAAATGAAGTGCCTTGCTTTAAAAACTCTTATCTTCATTGCAAAAGCTTCAAAGATGAAGGTTGAGGATATGAGAGTATTATTTGATGAGGGATGGCAATCTATGAGGATGAACTATTATCCACCTTGTCCAGAGCCTGACCAAGTCATGGGTCTTAGCCCTCACTCAGATGCTCATGGAATCACATTCCTCCTCGAAATCAATGAAGTAGAAGGTCTCCAAATAAAAAAGGATGGCAATTGGATGCCAGTTACACCACTGAATGATGCCTTCATTGTCAATATTGGAGACGTCTTGGAGATTCTGACAAACGGAATATATAAAAGCATAGAGCATAGAGCAACCGTGAACTCGGAAAAGGAGAGGCTTTCAATAGCCACTTTTTTAGGACCTGTATTGGAAGGTGATTTGGGACCAGCTCCAAGCCTAATCACTGCTGCTACACCTCCAAGATTTACAAGAGTAAGTGTTGCAGATTTCTTGAAGAACTTTTTTTCGAAGGAACTAAAGAGCAAATCGCACCTTAAGCAATACTATCTATGATTTATTATATCTAGGTCTGTATTAAAAAAACAATGTGTGTGTATCATATAAATAATGACCGTATGCTTTGTCTACTTAAAATGGCTTTTATGATTTAAGTGGGAC

>CL3969.Contig2_All

GTGAATTAGAGGGGTTCGGAATGGCTATTTTGGACAATTTCCTTAAATGCTTGTTGGTTCCAAGGTTTTCTTTTTTGAGGTAAGGTTTACAACAAACTTATTGTAGCTTTTTTATGAAAGAAAAGTACAAAATTCCACACTATGTTTACATATTTCTTACATAAAATCGTTATTGCAGCTAGGCGGGATACCAGCTGGGACCGTTGGGCTTGGAAGCATCGGTACCGCCAACACGTATAGCTCGAAAAAGAAGTCACAAATCCACTATTCCTTCCAACTTGAGAATATGCTGGTGCAGATCTTGTTCGGAGGTGAGTTATTTTAAAAAAGTTTATTCATAACGACAATAACATATATGATTATATGCATAATTACTCGATAAATAAACACATAACACTGTTCACTCAACATAATACAATTATGTAAACCATGCAAAACCGAGTGACTTTAACATCATTTAGGATCAACTGCATCATCGTTCTTCCTGAACAACTTGTGTTCCATATGTTGTTGGAACGTCCGAGGCGGAAACCGTGGTGGTTCCGCCTCGGAAACAGTCTCCGGTAGCGGTTTCAGGACGATCTTCTCCTTGGGCGGTTCACAGAAAACCGCCCAAGAAATCCTAACCTTCTCCTTGTTCACAAGCCCTCTATGGAGGATGCTTTTGTACTTTCCATTACTTAGGATCTCAATGGTGTCACCAATGTGAAGGATGATGGAGTCCGGCACGCACTGTGCCGACACCCATCGTCCGTCGTAAAAGACTTGGAGCCCTGGGACCATGTTGTGGAGGATGAACGTGAGAGCGCTCACGTCTGTGTGCGCCTCGACACCAAGCGCTAGCTCGGGTTGAGGGCATTTTGGGTAGTAGTTGATTTTTAGTTGGAGGAGTAACTCATCGGTCCCTCCGACCTCCTTCTCGAGCCTCCCCTCGTCCAGTCCCAACCCGAGAGATAAAACCACGAGTATCTCGGTGGCGAGTGCTCGTAGTTGCTTTGCGTACTCTGCAGTCGCCGGGATGTAATCTTCCGGCTTCGTTGGCCATATGGTCAAGTCGCGCTTCTCCTCCGGGAAGACGAGGTGAAAGAAGTAATCTTCCCATTCGAGTTGTCCGCAAGCATTGTTAGCCAATTTACTCCCGTACCCTTGCCTGTTCCCGGATGCGATATCGTTAGCATACTTCTCCTTCTCCTCCACGGGCTGACCGAAAAAGCCCTCCCCCGCGGCCTTAACACGGTCGATCACGTCTCCGGGGATCCCGTGGTTGACGACGTGCATGAAGCCCCACTCGGTGGCGGCCCTCGTGAGCTCGTCGCGACACTTTTGTCGGGTCTCCGGGTCGTCGGAACGGATGCGGCTTAAGTCGATGATCGGGAGTTGAGGGGCGTTTTCGTGGTGGAAGATGTTGGTGATGGTTTTGAGCTCGTGTTGTGGGCGGATGTATTCTTTTGGGATTTGGTGAATCCCACTTGTGGCTAAGCTTTCGACTCTTGTGTTCGTTGAAACCACCATTTTTGGAGTTTTTTTGTTTCTGTGGTGGTGGTGGTGGTGGTGGTGGTGGTGGTGGTGGTGGTGGTGTTGTGGAAGGTGGTTTTATAGGGAAATTAGTGAAGTTGGAAAGAATGGTGGGTGGTGAAGAATGGTGGGTGGGTGATGGGGTTGTTCTTAATTTTATGGTTGGTGGTTTGACTAACGGTCAAAGGACTTAAAGAAGGCACAAGTGAAG

>Unigene15127_All

TCTCTCTAGATCTCTCTCTCTTTCTCTCTCACACACACACTTCTCTCTTTCTGTGTGCAAATTAATTAATATGGGTATCAGAGGTATGCCTTTCTCAGTGAAGGTGATTGAGAAGGTGGTGGTGGCTGCAGAAGAGCCATGGGATGATCGCTGGTTACCCTTTACAAACCTGGACTTGGTTGTCCCTGCTTCTGATGCTGCTTCTTTCTTTTGCTACAAGAAACCCTTACCCTCCCATGGGAGTAGTAGTAGTTTCACCACCATGCTCAACACCCTTAAAGATTCCTTATCGCGAGCTCTCGCACTCTATCACCCGCTCGCCGGTGAGATCGCGTGGAATGCGGCTGCCGGAGAGAATCAGATCCACTGCAACAACCAAGGTGTTGATTTTACTCATGCCGTTGCTGACGTGCAGCTGAAGGAACTCGATTTCTACAATCCGGATGAGAGCATTGAGGGTAAACTAGTACCCGAGAGGCTACGTGGGGTGCTTGCTGTCCAGGTATATATGTAGTAAATTACGGTTTTTGTTTAACCAGATAATCGATCCTATAAAATTCTATGAAACATTCATATATTTAATATAAAATATATGAAAAGTTGATGAGTATGGAAATATGGATATATGAAAAGTCGATGATTATATATGGAACCCGATGAGAGAATATTGTGTGGGAAAAAAAGTTCAGACATGAATTGTAAGAATGGGATTTGGATCCTTAGATACTTTTAATCTCAGAC

>Unigene18529_All

GCCGGCCCTCGATGTACTGATGGCAATTGGTTTGAAAGGAAAGTCCACGGAAAGAGTGGTCTAAACACTACACATTGAAAATCCCAAGGTTGACCGACAGAAATTCAACATCGCACGCTCAAACCACCCACCCACCACTATCAACTCTCTAATGGCGGACTCCATCACCTCCTCCCAGTCCCATGTGGTGGCGATACCATACCCCGGCAGAGGCCACATCAACCCAATGCTCAACCTCTGCAACCTCATGTCCCTCCGCCGCCCTTCCGACCTCCTCATCACCGTCGTCGTCACCGAAGAATGGCTCGGATTCATCGGATCCGACCCGAAACCGACAAACGTCCGCTTCGCCACCATCCCTAACGTCATCCCGTCGGAGCTCGACCGCGCCTCCGACTTCGCCGGCTTCATCAAATCCATTCACACAAAACTAGTAGACCCGGTCGAGAGATTACTCCGCCGGATGGAAATTCCGGCGACCGTAATCATATACGATACCTACCTCATGTGGATCATAGATCTCGGAAAACGGTTGAACATTCCGGTGGCTTCCTTCTTCACGATGTCGGCCACGGTGTTCTCCATGTGTTATCATCACGATCTCCTCCTCCAAAACGGCCATGTCGGAGATGATTATTTCTCAGAAAAAGGTGAGGAAGTGATCGATTACATACCTGGAGTGCCTCCCATGCGCGTGGCTGATCTCGTGACAGGCTTCAATGGCAAAGGAAAAGAGGTTTTTCCGTTAGCTCTGCAAGCTATTTTAATGGCGGACAAAGCTCAGTTTCTGCTTTTCGTGTCGGTTTACGAGTTGGAAGATAAAGTGATCGATGCCTTAAAATCGGAGCTTTCGGTACCCGTTTATGCTATTGGGCCGTCCATTCCCTACTTCAAAGTCCAAAATGACGAAAACACCCCTGTATGTGAAAATGACCAAAATGGTCGTCATTATGAAAATGACCAAAATACCCCTGGCTATTTGGAGTGGTTGGACCGTCAACCCGAGGGTTCCGTGTTGTACATCTCGCAAGGGAGTTTTCTCTCGGTCTCGAATGCGCAGTTGGAGGAGATCGTGGCGGGTGTGCATGAGAGCGGCGTACGGTACATGTGGATTGCACGTGGCGAGACGTCTCGGTTTAGACGTGAAAATGACGAAAAGGGGATCATTATACCTTGGTGTGACCAATTACGGGTGTTGTGCCATGGTTCGGTAGGGGCGTTTTGGTCACATTGCGGATGGAATTCAACGAAAGAAGGTGCATATGCGGGGGTGCCGATGCTCACGTTTCCCATATTTTGGGATCAAGTTCCAAATAGTAAGATGATCGTTGAAGATTGGAAAATGGGACGGAGGGTGAGGGTTGACGAGGGTATTTTGGTCACTCGAGATGAAATTGCGAAACTCGTAAGGGGTTTCATGGATGAGGAGAGCGAAGAAGGTAAAGAGATACGCAAAAGGGCAAGAGAAATAAAGAAGATATGCCGACAAGCGACCAATGAAGGAGGGTCCGCTCAAAAAGACATCGATTCATTTATCAGCAATATCTTGAACAGTCGAAACAATTAATTGATGAAGTATATTATAAATGTACTTGACGATATTTTA

>Unigene62812_All

CATGGTTAAGGCATCCACAAGTTGATCTTGGCTGGGGTCGGGTAGTAGCACGGTTGCCATGAGGTGGTAGAACAACGTTTGAGTGGAGGTCTTTGAAGGTTGGACAATAAGCAAGAAACATGGCATTTCTCATTACACCATTGGCACTTTCCTTCAAAAGGGAGGCGGCATGGAGGCGGCAACCCGATTTCAGAAACTAAGATGGTTCTTCAGCTCGTTTTGGGTCTTCCTAAAGGCGACTATGACACCATCGCAACCTTCATTTAACAGTCTGATCCTCTTCCAACCTTTACCTGCTCAATCCCAACAGTCTATCGTTCCCTTATTTATCTGGAAGCTTGTATTGTAACCTTATAAATACTTTGCTTTTGGAAGGAAAAAGAACAACAGATTCTACACGTTCTAACACTTATAAACAAATTAATTCACATTACATGAAAGCCTTCAACAGGATTAGAAAAGTATCATAAGCAATCCACAGGATAAGAAAAGAAAGCTACTTGCATATCTGCTCACAACTCAAATCCTTTTGTAATCGTTAATAATGATCTAAATTTGTCACGAGTTCACCAGTGAAAACAACGATCGAAGCTAATCATTTTGCGATTTTGGAATGTCATCGATTTCGTCGGCTTAAATGTAGTCTTCTAAACCACCATTGAAGATCGAAACAAAATCCTTCATCTCGGCCGCTGAAAGACACACACCAATTTCCAAATCTTCACTCGATTTGCTACAGGCGTTCAACGAAATAGATCCACTGTAATCAATCGAAACAATCTCATACTTTTTCGGCTTCCCCCATCCAAAATCAGCGGTATCATAAAACTTAATCCTTGGCGTTCCGGCCACCCCCGTAACCGATAACGGCTCCTCCCCAACCAAAAACTCGAAGTCGAACCAACTCTCGGCACCTTTCAAGATCCCGTCTTTATCGCTCAACTTCTTATGTAAACTCTCGCCAAGCAACTTGGCCGCTGTAACGAAGCCGTCTTTATTGGTTAACTCACTCTTTCTAGCGAACGCCCCACACGGGGCAACGCAGTTGCCCGAATACGTCGAGGGGACCACCGGAACCAGACGCGACCTGCAATCGATCACGAAACCAAACACTTGTAGCTCGACGTCGCGAACTTTCGCCAGCGAGCACCATATATACGCGCATGCGACCGTAAAAGACGATACGTATTGCAACGACGGCAGTTGGGTCGATACCCGTTTCTTTAACCGGTTAACGATCGTTCGGGTCAACACGAACGTGGCCCGAACGTTGTTTGTTGGTCCGGAAAGGCTCGGAGGCCGATAATCTTGAACGAAAGTTTCTAACTCTGCTTTCTTTAGATATACTTCGTCTAGAGTCGGGTGTTTGATCACTCTATCGTACAACGGCAAGGCTCCGTTGGCTAGAAACGACTCGTGGGTGCCGGATCTCGCGATCGAAGTCCACGCGTCCATGAAACAAAACCGGGTGCTGGCATCTCCGAGACAGTGATGGTTTGTCATCCCGATGGAGATCCCAGCACCCGGGAAAACCGTCACCTGGACCGAGAACAGCGGGATCGATAGGTAACCCTCGGATGCCTTTGTGGCATCCCTGAGAAGTGGTATAAGAGGATAAAATTTATCGCAATCTCGAGGATGGTTTCCGATTAGATCGTCGAAATCAAGATCGCTCTCCGCGATGGTAACGACTACGTAGTCGCCATCCACGTGACGGATTTCGGGTTTTCGAGCAACGCCTGAAGGGTTTTGAGCGGGATACACGATCAAGTTACTTGCAAACGGGAAAAAGTGTTGGAGGGTTATCGATAACGAGTGTTTAAGGTTCGGAATAACGGTTTCGACGAAATGGGATTTAGAATGTGGGAATTCGTAGAAGAAGAGCTGGTGGATTGGATGGAAGAGTAGCCATCCCAGATCGAAGAAAGTAAGTGGCAGTGACCTTTCGCCGACGGTGTTCGGCGGTGGCGAGATCCGGCATTTCTCAACAACACTTAACAGCTCATTGGTGGTGGTGGCAGCCATTTTTGGGTTTTTATTTGCTTGTGGCTAGAAATGCTTCTTTGTGTAGATATTAAAGT

>CL285.Contig2_All

ATGAATCATATATGATTTTGTACGTGATTTTTGTACTTGAATTACATATGATTTTACATAATCGTATATGATGGGCAAGACTTTGGGCGGGCCACCAAAGCGGGTTTAGCCGATTAGAGCTCAGTTACAGGAACAGCCGTCTGATGGAAAACGACTTTCGCATTAGGTGCTTTATGAAAGCCGGTCCACAGCGGTGCATCGCCTATTTCTCGTTCTCGCTCGGGAGCCAAAACGAACACGCCTGCTACTTACTACGTCACCCTCACATACACCGGGAAAAAGGTTACGATAGCAAGCCCCTCCCCGGCCGGAAAGGGCTTGCTGATAGAGCTGGGCGGGGAGGCGATACTAGTTATCAAGTCAGAGAGAGGTTTGGCCCGTAAGCTGGCCCCCCGGGAATCTTTAGAATTACCACTGCTTAGCTTGATATATACGTGATTTTGTACATGAATCATATATGATTTTGTACGTGATTTTTGTACTTGAATTACATATGATTTTTATACATGAATTATACGTGATTTTGTACATGGACGACCATTCTCACGTTCTCGTAAATAGTTAGCGATCTCGTTTGGACCTTTTTTCTATATATATTATGTGTGTGTATATATATGATTGATCAAAGCTAAGGTTCACAAAGTAGGCATCATCCTGAGTTCAATCAAGGGGTCATTAGAAGAAGGCCACAAGATGTCGAATGAAGAAGAAGGGCTGCAAAAAATGAGGGAAATACGTTCTGCCATAGTTCTTCCAATGATCATCAAAACCGCCATCGAACTTGATCTCTTTGAGATCATGGCCAAGACTCCAGGTGCCCGTTTCTCTTCGTGTGATCTTGCTTCTAATCTACCTTCACAAACCCCACAAACCCCACACTTACTCGAGCGCATTCTTCGGTTTCTTGCCACCCAATCCATTCTCAAATCGACCACGGAAACCGATGAACACGGGAACTCGAAGAGCTTGTATAGCATGACACCCGTGTCGAATCACTTTGTTCGCGATCAAGATGGGACTTCTTCGGGTTCATTACTTCTCTTGACCTATGATAAAGTGTTTTTGGATTGTTGGTAATTGTTCTCTAAACTTCATACCTTACATCTAGCTTTATAAGTGAACTTAATTGCATGTTTCTCTCTTTGTATATATATGTACAGGTTTTGTAAATTATAGAGGTTGTAAAGCATGTGGCGATACCCTGACCATGGGTCGGCTTTCAGGTCGCTCCATGGCCGCCGAGAATTAGTCGGGTGCCTGTATGGATCCCGAATACCTCGTCGAGTTCATGTCGAGACGTGTGCGTGAGTTATCTGTCGTTCAAAAAAAATTCTTATTAGAAATCTAGTTGGCACCTTGGTGGTTTAAAGTTGGCATCGTTTGCATATAATTGAGCTTGAAACTTGAAGCTTGAATATCCCATTAGTCTCCAATAGAATATGCCCCCCACTGTTGCTGCCTGTTGACCGTTTGGAGTTTAACCCACGGAAGAAAGAAAGAACAAAACCGAAACTTTAGGACAAAACCACCACCACCTGAGCTTATACAATTCGATCGAGCTGGATATGGAGAAAGTGATCCGAATCCAAAGCGTTCGTTGAAGAGTGAATCATCGGATATTGAAGAACTTGCAGACCAATTGCAGCTTGGATCTAAATTCTATCTCATTGGTGTTTCTGTGGGATCATATCCCACATGGAGTTGCATCAAGAACATACCTGAAAGGCTAGCAGGAGTTTCTTTAGTGGTTCCATTCATAAACTACAGATGGCCATCACTTCCTGATGATCTCATCCAAGATGATTACCGGAAGAATCTTGCCAGGTGGGCGGTCTGGATTTCGCGCCACACCCCCGGATTGCTACACTGGTGGTTGACTCAAAAGATGTTCCCTTCATCCTCTGTTCTTGATAGAAACCCTAAATTCTTCAGCACTAAAGACTTGGAAGTCCTGAAAAACACTCCTGGATATCAGTTGCTCAGTAAGAGCAAGCTGAAAGAAGAACCGATCTTCCATAGTCTTCGTAAAGACTTCATGGTGGCATTCGGGAAATGGGATTTCGACCCGTTGAGTATGAGCAATCCGTTTGGTCAAAGTCAAAGTCAAGTCCACATTTGGCAAGGTTACGAAGACAAGGTTGTCCCGGTCGAACTACAAAGGTTTGTTTCGAAAAGGTTGCCATGGATCAAGTACCATGAAGTTGGTGATGGAGGACATCTGCTTGTGTATGATAGTGATGTATGTGAAGGCATTTTGAGGTCTCTTTTGCTTGGAGAAGATTCTCCTTTGTATAAACCTAAATTTGATTAAAACTAGCTAGAATGTCCCTTGTAAATTAATCATATGATTGTTTAAGAAATGGATCATATTTATGGTTTGATTTTATTTAATGTAACTCCT

>CL10851.Contig1_All

TCTCTCTAATTGGGTCTTCCAATTTATGCCCACTTCTCTAATCTCTAGTCTCTGCTTTTGTCCATTCAGACGATATGGGATACATCCATTTCTTACCAAAAGGGGGAATTAATAAATCTGAACACTCAGCAGGCATCCTGTGAATTAAAAGTTAAAGAAACCAAATTTGCTATCCCATTTTTCACCGTTAAAATACTCTTTCCGTAGAAGGTTTGTGTGCCTATAAACTTTAATCACAACAAGCAAATAATATTTATTTGCTTCTTTTCAAGAACTATATAAGATCCATGATACTTACGTAGAGATATGATCGTATTAAAGGTATTAAACCAATAAGCTGATGAAAAACTGTTTCAGCCTCCATTGACATTGTAATGATATTGTGTCTGGGAAAGGTTTTAGGAATTATAATCATCCAGTTTAGATATCATACAGGAACTTGCTGAATAGCTTCATGTGTTCCGCTTGCAGCGAAATCGCAACCGACAAGCTTCCGTCATTAATCGGGCTCGGTAACACGAAACTCAACCCTTCATACGCAATCCCACCGGGCCCCATGAATATCGGCCGACCCCATCCGAAGTCGGCATCGTGAATCGGGAGTCTGGCCCAGCTCGTTATCCCGAGATTTGGGCATTTAAAAGTATGGGCCCCACGAACCAGGGCCTTCAGATCGGGCTGCAGTTCCAAGAAATCGAGTGCCGACTTTAGATAATCGTCGTCCATCCTTGCCAACGCGTCGTGGATTTTACTAGCAGCATACCACGTCGGCTTCGACTGCAGGTCGCCGGCCACCGCTACCGGGGTGGTCGTGAATATGACGTTCCCGAAATAGCCCGGCGGCAGCGCGGGCCGGAGGCGGGCCCGTCCGTCGGTCGCGATGTAAAGCTTCGTGTCCTGGTCGTCCGGAAGGCCGCGCGCTAGGCACACGCACCTCCAGACGTGGCCCGAGAGCATCTCGTAGGAGCTGTAATTGACGGTGTTGCCGGCTTCTTTCGACTTCCCTTTGAGTGCGTTGAGCTGGTCTCGTGTTAGCTTAAAAATCGAGACCGCGGTCTCGGGAACCGATTGGTCGTCGGTGGGTTTGGGTGCGGTTTTCATCGGGGGAGCGGGTTGGTACTCGATGTGGTCAAAGGCCGGTCGGGGCGGGTCACGGGCACGTAGAAGGGTTCGGTCGATGAAGGGTGGGAGGGTTAGGTCAAGGCCACGAGCCATATCGGACCAGGTGTTGATGAAGTGCAGCCCAGATGCACCATCAGCCGCGTGATGTTGCATCCCAACTCCTAGCGACACCCCACCACATTTAAAATAAGTAACCTGCAAGACTAGCAGCGAATAGGATTCAATTCCTTGAGTGTAATCAACCGCCGGAATAAGCTTCCGGAGCTCCAACGTCGGTGCAAAATCACCGAAATCATCAACCACGCCGTCGGACTCCGCCTCCACGAACAACACGCCTTGTCCTTGGCAATCGATCTCGATCCGGCCATCTTCATCTCTCTTCAACCGTCCTCCCATCGGATAAAACGGAACCAACGCCTTGCTCAGCGCCTCCTTCATCACCTTCGGATCGAAGAAGTTCGCAGCGCCGGTCGGCCGGTAGAAATACACACTCGGTGTGTGAAAATTAGGGACAACCAGATCGACATTCGAGTTCCATAGATTTATCCTCGGCGTCTCCTCCGCCGGCCTCACCATCGTCGATTCTCTCACCTCGATCTTCATCTCCTTCTTTTGCTCCAATTCACTGTGAAGAAACGACAACAGATTTTGGTGCAGTGTAGGTGTTCGATGAAATGCGTGAGATAATTAGAAGAAGAAGAAGGTGGTGCTGGTGGTGGTGGTGGTAATATTGGTATGGTTGGTGTTGATTTGGTATGAAGTGGTTTTGAGTTGGGAAGACTTTAAAAAAGAAAGCGT

>CL18920.Contig24_All

CAAGTGTCGCGCTCTGGGCTGTCCTCACGTTCACAACCAAAAAGTGGTTCACATTTGAACCCACCCCATATAATTAATTGCATAAGTTTATTCTTCTATGATGATGATTAGATATGGATCATTTTAACGTATGGACACTTGGACAGTAATTCACTTGTATGATAGACTGAATGTTTCACTTGATACTTTTTCTTTATCGTGAATTCGTGATCCACTAAATCTATGGGATTGATACAGACCAAGATGGTACCCTTCATATTCATCTTTAAGCCACATATTCATGATCTTTCCTGATACAGACCAGTACGGTCCCTAGGCTGTCTTTTTCCATGAAGTGCACTTGTTGCTAGTTGAAAACGTTTGAACATTGAATAAGACATTCCTACATGGTGCCTTTCCACCATTCCTTAGTCTAAGGTGCACTGCACAAATACACAACTCTAATGTCCAATATCAAGGGTAGATTCATCGTAATAACTTGCTATTGGGTGTCATTCTGATTGATGATCAATTAAATAACTAGAGATCAAAAAATTTCAAGACTACTGGTCTCAACATACTCAGATTACCAGCATGCACAAACACCTGGTCTTCAACACTACAGGAGGATTTCCAGGACCACATATAGAAAAAAGTGAAAAACGATCCGAGCTGACATGACAAATAATAGTTATGACAGAGTAACGAAAAAAGATAGCAAATCTTTCTTCTCTTGTTTATGTAAAAGCCAACTGTTCTTGCTGTACTGGAATCCAAGACAAGCCTACCCCACAAACACACACACACACATATTGAAAGCAAATTTTTTATAGTATACCATACTCTGTCTCACTTGCACAGACATGCATCACTTCAACTCAACGGAATACCAAACGTAAGTCACAGCTATGCATCTAGAACAGCGGAATTTATATGATTAAGCAAGAGTCTTATATGTCATTCTTTTTCTCTTTAATGTACCTTTTAGTGATCCCGAATCAAGATGGAAGACGGTTTTCATCCACCTTGATCCATTCAATAAAATTGCTTAGATTCTTTTCTGAGCTCCCGCCTTTTCTGATGCTACTCACAGCCTTTTCTTTGAGAGCCAGGGCGTTATCTTTGAATCTTGTGTTGTTGAGCAGCTTGTTCACTTTACTTTTGATCTCTTCTCCTGTGACGATTCCTCCTATGTTTTTCTTCAACGCCAGCCCGTTCTCCCAGATGTCACAAATGTAAGTTTCGTTCTGAAATTGATCAGCAAAATACGGCCAACACATGAAAGGGACTCCATTATTGACACCTTCCAGAGTAGAGTTCCAACCACAGTGGCTCATGAAGCAAGCCACCGAAGGATGAGATAGGACCTTCTGTTGAGGTGCCCAGCTTACGATTCTTCCTCGACTGCCAATTCTATCCATAAACCCTTGAGGCATCTATCAACGTCTGAAAGCTCAAAATTGAAGCCAATGTAACTGCTGAGGCAGGCCAGAATGATGCCCTTCTAATTCCCATCTTCTCTGCAACAGGCATGGTTTGGAATATTGCTTCGGTCTGCTTAGCAAGGTCGTTCCTGTCCTCCCATGGTTCCAACCCATCTGGGAGCGAAACCATTTGCACAAGATCACCGAAACCATCTTTTTCCAACCAGGCGCTTGCTACCAGTTTATGTGTGACCTCTGTGTTTATAAACGTGACTTTGATACCCTGCTTAACCAAGCATTGAGCAAACTCCATCAGAGGAATTACATGGCCCTGTGCTGGATAAGGTATGGCTATAACATGACTATTCTCCATATTTTTGTGTTGGTGGTTACTTCTGGATTAATCTAACTCTTATAGACGGCCATGGCTGAGATACCAAAGCTCCAAATCTTGTCTGTTATATGTGATCTATGACAGAATTTGATCCTTTGGCCTAGTTAGTCCATGGAGGTAACTTCAGGATTAAACAGTTGCAACTGTCTTACTTAGTGAA
