# Supplementary material for: Transcriptome analysis of flavonoid biosynthesis in safflower flowers grown under different light intensities
Source: PeerJ. 2020 Feb 21;8:e8671. doi: 10.7717/peerj.8671 (PMC7039124; doi:10.7717/peerj.8671)
Supplement: Supplemental Information 6 [file peerj-08-8671-s006.docx]

| Ingredients | Sample | Content (%) | SEM |
| --- | --- | --- | --- |
| Total Flavonoids | HL | 4.547 | 0.567 |
| Total Flavonoids | ML | 4.876 | 0.989 |
| Total Flavonoids | LL | 4.373 | 0.885 |
| HSYA | HL | 1.73 | 0.2245 |
| HSYA | ML | 1.9 | 0.0419 |
| HSYA | LL | 1.91 | 0.3729 |
| Kaempferol | HL | 0.079 | 0.00401 |
| Kaempferol | ML | 0.121 | 0.00138 |
| Kaempferol | LL | 0.07 | 0.0045 |
