# Supplementary material for: Transcriptome analysis of flavonoid biosynthesis in safflower flowers grown under different light intensities
Source: PeerJ. 2020 Feb 21;8:e8671. doi: 10.7717/peerj.8671 (PMC7039124; doi:10.7717/peerj.8671)
Supplement: Supplemental Information 7 [file peerj-08-8671-s007.docx]

| Gene | Sample | Relative Quantity | Relative Quantity SEM |
| --- | --- | --- | --- |
| *CtHCT1* | HL | 1 | 0.36745 |
| *CtHCT1* | ML | 1.08 | 0.22919 |
| *CtHCT1* | LL | 0.40635 | 0.19617 |
| *CtHCT2* | HL | 1 | 0.37364 |
| *CtHCT2* | ML | 0.4974 | 0.16334 |
| *CtHCT2* | LL | 0.095 | 0.06212 |
| *CtHCT3* | HL | 1 | 0.57149 |
| *CtHCT3* | ML | 0.09401 | 0.01909 |
| *CtHCT3* | LL | 2.33197 | 0.93153 |
| *CtFLS1* | HL | 1 | 0.26817 |
| *CtFLS1* | ML | 0.95757 | 0.21791 |
| *CtFLS1* | LL | 2.58683 | 0.46986 |
| *CtFLS2* | HL | 1 | 0.35364 |
| *CtFLS2* | ML | 1.09409 | 0.82894 |
| *CtFLS2* | LL | 0.60329 | 0.24878 |
| *CtANS1* | HL | 1 | 0.14759 |
| *CtANS1* | ML | 0.77384 | 0.1011 |
| *CtANS1* | LL | 3.91551 | 0.73634 |
| *CtANS2* | HL | 1 | 0.13179 |
| *CtANS2* | ML | 1.34067 | 0.39321 |
| *CtANS2* | LL | 0.47517 | 0.16659 |
